# Serology study after BTN162b2 vaccination in participants previously infected with SARS-CoV-2 in two different waves versus naïve

**DOI:** 10.1038/s43856-021-00039-7

**Published:** 2021-10-13

**Authors:** Luca Dalle Carbonare, Maria Teresa Valenti, Zeno Bisoffi, Chiara Piubelli, Massimo Pizzato, Silvia Accordini, Sara Mariotto, Sergio Ferrari, Arianna Minoia, Jessica Bertacco, Veronica Li Vigni, Gianluigi Dorelli, Ernesto Crisafulli, Daniela Alberti, Laura Masin, Natalia Tiberti, Silvia Stefania Longoni, Lucia Lopalco, Alberto Beretta, Donato Zipeto

**Affiliations:** 1 Department of Medicine, University of Verona, Verona, Italy; 2 Department of Infectious, Tropical Diseases and Microbiology, IRCCS Sacro Cuore Don Calabria Hospital, Negrar (Verona), Italy; 3 Department of Diagnostics and Public Health, University of Verona, Verona, Italy; 4 Department of Cellular, Computational and Integrative Biology, University of Trento, Povo (Trento), Italy; 5 Department of Neuroscience, Biomedicine and Movement Sciences, University of Verona, Verona, Italy; 6 Division of Immunology, Transplantation and Infectious Diseases, San Raffaele Scientific Institute, Milan, Italy; 7 Covi2 Technologies Srl, Novara, Italy

**Keywords:** Medical research, Vaccines, Virology

## Abstract

**Background:**

The antibody response to SARS-CoV-2 mRNA vaccines in individuals with waning immunity generated by a previous SARS-CoV-2 infection, as well as the patterns of IgA and IgM responses in previously infected and in naïve individuals are still poorly understood.

**Methods:**

We performed a serology study in a cohort of BTN162b2 mRNA vaccine recipients who were immunologically naïve (N, n = 50) or had been previously infected with SARS-CoV-2 (P.I., n = 51) during the first (n = 25) or second (n = 26) pandemic waves in Italy, respectively. We measured IgG, IgM and IgA antibodies against the SARS-CoV-2 Spike (S) and IgG against the nucleocapsid (N) proteins, as well as the neutralizing activity of sera collected before vaccination, after the first and second dose of vaccine.

**Results:**

Most P.I. individuals from the first pandemic wave who showed declining antibody titres responded to the first vaccine dose with IgG-S and pseudovirus neutralization titres that were significantly higher than those observed in N individuals after the second vaccine dose. In all recipients, a single dose of vaccine was sufficient to induce a potent IgA response that was not associated with serum neutralization titres. We observed an unconventional pattern of IgM responses that were elicited in only half of immunologically naïve subjects even after the second vaccine dose.

**Conclusions:**

The response to a single dose of vaccine in P.I. individuals is more potent than that observed in N individuals after two doses. Vaccine-induced IgA are not associated with serum neutralization.

## Abstract (web summary)

Dalle Carbonare et al. perform a serology study in participants with a prior infection of SARS-CoV-2 and those who are SARS-CoV-2-naïve, who received two doses of the Pfizer-BioNTech BNT162b2 vaccine. After a single dose they observe a quicker recall of pseudovirus neutralization titres in previously-infected participants and a potent IgA response in both groups that was not associated with serum neutralization titres.

## Plain language summary

Antibodies are proteins produced by the immune system that are released into the bloodstream and help fight infections. To understand how the immune system responds to COVID-19 vaccination in individuals who have had a previous SARS-CoV-2 infection, we compared the types and levels of antibodies produced after first and second doses of the vaccine with those of individuals who had never been infected. We found that one dose of vaccine, even several months after the infection, was sufficient to boost a very efficient response by eliciting specific types of antibodies, some of which were and some that were not able to neutralize the virus. We also observed an unusual antibody profile in almost half of individuals who had not been infected. These findings may help better understand the immune response to COVID-19 vaccines.

## Introduction

As we write, four COVID-19 vaccines have been authorized for use by FDA and/or EMA, and additional vaccine candidates are under evaluation. The authorized vaccines are based on the use of mRNA^1^ or adenoviral vectors^2^ that induce the expression of the SARS-CoV-2 spike protein. Apart from the Johnson & Johnson adenovirus-based vaccine that requires only a single dose, all other vaccines are based on a double dose regimen to maximize their efficacy.

Increasingly available anti-SARS-CoV-2 antibody and virus-specific T cell data support a strategy that previously infected (P.I.) vaccine recipients have sufficient immune response from only one vaccine dose^3–6^; this would have a substantial impact on global vaccine supply. Spike-specific IgG antibody levels elicited by a single vaccine dose in individuals with prior SARS-CoV-2 infection (P.I.) were similar to those seen after two doses of vaccine in individuals without prior infection (naïve)^7^. In another study, the antibody titers of recipients with preexisting immunity were ten to 45-fold higher than naive recipients at the same time points after the first vaccine dose of vaccine, and no increases in antibody titers were observed in P.I. recipients who received the second vaccine dose^8^. Similar data were reported by Bradley et al.^9^. Interestingly, in the same study naïve recipients, at baseline, exhibited a significant level of reactivity to the S2 subunit, suggestive of a preexisting cross-reactive response to common coronavirus infections^9^. Rapid kinetics of antibody binding to a trimeric spike protein and live-virus neutralization assays was similarly observed in a cohort of P.I recipients who had received one dose of an mRNA vaccine^10^. In addition, two of these studies reported that vaccine reactogenicity was more prominent in P.I. individuals after the first dose but similar between the two groups after the second dose^7,8^. A cautionary tale for the use of a two doses regimen in P.I. individuals was raised considering the possibility of antibody-dependent enhancement^11^ or antigen exhaustion as a result of an over-boosting of immune responses^12^. The lack of an established correlate of protection against disease and/or infection adds further complexity. The emergence of SARS-CoV-2 variants is also a concern, and Stamatatos et al. highlighted the importance of two dose regimens in both naïve and P.I. individuals to achieve cross-variant neutralizing antibodies^13^. An additional component of the immune response to SARS-CoV-2 that could influence the outcome of vaccination is the presence in most SARS-CoV-2-naïve individuals of variable levels of preexisting immunity to spike protein epitopes that are shared with other common human coronaviruses (hCoVs)^14,15^; these have been suggested to be potentially protective or pathogenic and may shape the kinetic and potency of the immune response to the vaccine^16–19^.

Here we report data from a serological profile of a cohort of 101 naïve and P.I. recipients who received both doses of Pfizer-BioNTech BNT162b2 mRNA vaccine. We took advantage of the availability of two different subgroups of P.I. recipients who experienced a SARS-CoV-2 infection during the first wave (Spring 2020) and the second wave (Autumn 2020) of the pandemic in Northern Italy to investigate the different effects of vaccination in recipients with recent-active or past-waning antibody response. We observed that one dose of vaccine is sufficient to stimulate an efficient response, eliciting both IgG and IgA antibodies, but only IgG, and not IgA, are associated with neutralizing activity. In addition, almost half of the vaccinated naïve individuals show an unconventional antibody response, producing IgG but not IgM.

## Methods

### Study population

We analysed the sera of 101 health-care workers with and without preexisting SARS-CoV-2 infection (as per former nasal swab positivity) who received their first vaccine dose (BNT162b2 mRNA, Pfizer-BioNTech) in January 2021. The study was approved by the Ethics Committee of the University of Verona (approval prot. N. 1538) and of the IRCCS Sacro Cuore Don Calabria Hospital (approval prot. N. 50950), and samples were stored in the biobank of the Department of Medicine, University of Verona, and in the Tropica Biobank of the Don Calabria Hospital. All relevant ethical regulations were followed, and all participants provided written informed consent.

### Serologic assays

The SARS-CoV-2 IgG-N assay and the SARS-CoV-2 IgM-S assay (Abbott, Ireland) are chemiluminescent microparticle immunoassays (CMIA) used to detect IgG antibodies to the nucleocapsid protein and IgM antibodies against the spike protein, respectively, of SARS-CoV-2 in human serum. The automated assay was performed according to the manufacturer’s procedure, using the ARCHITECT I System (Abbott). The resulting chemiluminescent reaction was measured as a relative light unit (RLU) by the system optics. The RLU of the sample (S) was automatically compared with the RLU of a specific calibrator I, resulting in an assay index (S/C). As per the manufacturer’s instructions, the interpretation of the results were as follows: index (S/C) <1.4 = negative, index (S/C) ≥1.4 = positive for IgG-N, and index (S/C) <1 = negative, index (S/C) ≥1 = positive, for IgM-S.

Serum samples were tested for the presence of SARS-CoV-2 IgA using the Anti-SARS-CoV-2 ELISA kit (EUROIMMUN Medizinische Labordiagnostika AG, Germany). The assay has been routinely used and validated for almost a year in the Laboratory of Neuropathology of the Department of Neuroscience, Biomedicine and Movement Sciences of the University of Verona (sensitivity 96.9%; specificity 98.3%). The assay detects IgA antibodies in serum binding the S1 domain of the SARS-CoV-2 spike protein. Samples were tested and analysed as recommended by the manufacturer, and results were reported as a ratio based on sample OD divided by the OD of the calibrators. Antibodies were considered undetectable (negative result) if the ratio was <0.8, borderline (inconclusive) between 0.8 and 1.1, and positive if >1.1 (LLOQ 0.26; ULOQ 8.75).

The SARS-CoV-2 IgG II Quant assay (Abbott, Ireland) is a CMIA used for the quantitative measure of IgG-S(RBD) antibodies (including neutralizing Abs) in human serum. The automated assay was performed according to the manufacturer’s procedure, using the ARCHITECT I System (Abbott). Results were reported as arbitrary Unit (AU)/mL, according to the following interpretation: AU/mL <50 = negative, AU/mL ≥50 = positive. According to the WHO International Standard for anti-SARS-CoV-2 immunoglobulin binding antibody units (BAU), the AU/mL are converted into BAU by the equation: AU/mL × 0.142 = BAU/mL.

### Pseudovirus neutralization assay

Lentiviral particles pseudotyped with SARS-CoV-2 spike were produced in 10 cm plates seeded the day before with 3 million HEK293T cells in 10 ml of complete DMEM, supplemented with 10% FBS. Cells were transfected using the calcium phosphate technique with 15 μg of an Env-defective SIV-Mac239 provirus construct expressing GFP in place of Nef^20^ and 1.5 μg PCDNA3.1 expression vector encoding the WT SARS-CoV-2 spike (reference sequence Wuhan-Hu-1, accession number YP_009724390) with a truncation of the C-terminal 19 amino acids. Supernatants containing pseudotyped virions were harvested 48 h post-transfection, filtered through a 0.45-μm filter, and frozen at −80 °C until used. Sera neutralization titers were assayed on Huh-7 cells engineered to overexpress the SARS-CoV-2 receptor ACE2 upon stable transduction with a lentiviral expression vector. Target cells were seeded on 384-well tissue culture plates 1 day before neutralization. The virus inoculum was adjusted to produce no more than 10% of monolayer transduction to ensure a linear working range of the assay. Sera dilutions were added to target cells using an acoustic dispenser (Beckman Echo 650) to reach the indicated dilution in DMEM with 10% FBS. The pseudotyped virus was then added to wells using a Tecan Evo^®^ 200 liquid handler. After 48 h, transduction was assessed by calculating the percentage of GFP-expressing cells upon nuclei counterstaining with Hoechst 33342 and measuring using the High Content Molecular Device Image Xpress^®^ Micro Confocal. Each serum dilution was evaluated in triplicate. Neutralization was measured by calculating the residual transduction activity of the pseudovirus considering the untreated sample as 100%. Fitted sigmoidal curves and IC50 were obtained using Prism (Graphpad) with the least square variable slope method and using the normalized dose-response protocol.

### Statistics and reproducibility

The CMIA tests were performed using IVD kits for Architect automatic system on a single replicate. The system is periodically calibrated according to the manufacturer’s procedures and quality controls are run daily. VEQ are run every 3 months. The Department of Infectious, Tropical Diseases and Microbiology is certified by Bureau veritas Italia for the diagnostic process according to the UNI EN ISO 9001:2015.

For neutralization assays, each serum dilution was evaluated in triplicate.

**Fig. 1:**
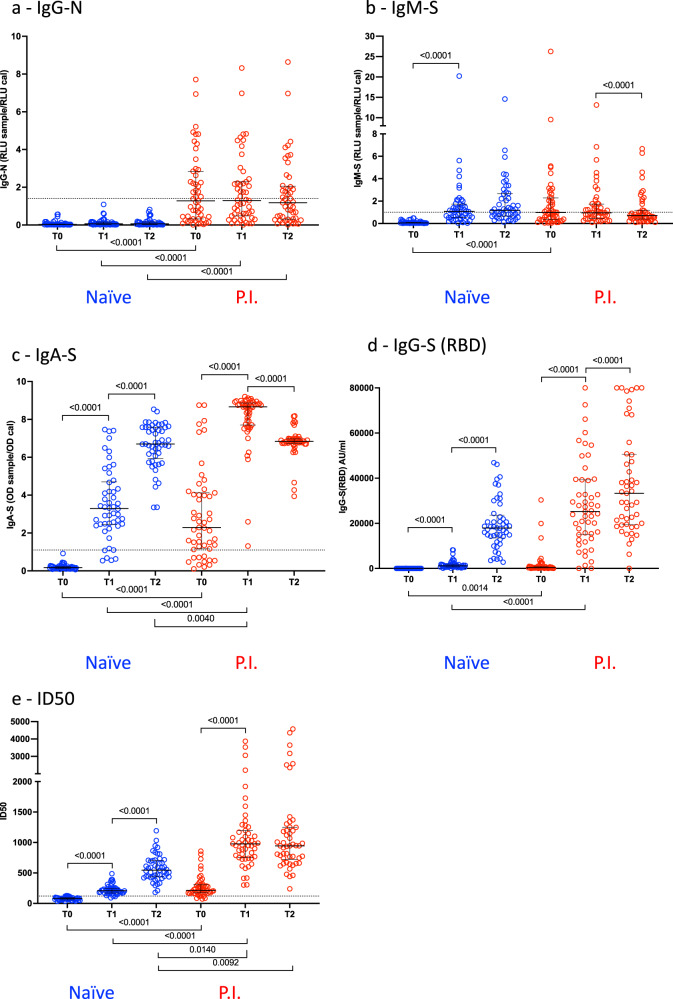
Analysis of the antibody response profile at the time of first vaccination (T0), second vaccination (T1), and 3 weeks after the boost (T2) in naïve and previously infected (P.I.) recipients. Median values with the interquartile range are displayed. The horizontal dot lines indicate the cutoff value to discriminate positive and negative samples for each assay, according to the manufacturer’s instructions. **a** IgG for the SARS-CoV-2 nucleocapsid protein N (sample sizes: T0 *n* = 48, T1 *n* = 50, T2 *n* = 49 for naïve, T0 and T1 *n* = 51, T2 *n* = 49 for P.I.); **b** IgM for the spike glycoprotein (sample sizes: T0 *n* = 48, T1 *n* = 50, T2 *n* = 49 for naïve, T0 and T1 *n* = 51, T2 *n* = 49 for P.I.); **c** IgA for the spike glycoprotein (sample sizes: T0 *n* = 49, T1 *n* = 50, T2 *n* = 49 for naïve, T0 and T1 *n* = 51, T2 *n* = 49 for P.I.); **d** IgG for the receptor-binding domain (RBD) of the spike (sample sizes: T0 *n* = 48, T1 *n* = 50, T2 *n* = 49 for naïve, T0 and T1 *n* = 51, T2 *n* = 49 for P.I); **e** pseudoviruses neutralization assay, expressed as infectious dose (ID50) (sample sizes: T0 *n* = 48, T1 *n* = 50, T2 *n* = 49 for naïve, T0 and T1 *n* = 51, T2 *n* = 50 for P.I.). *P* values were calculated using the nonparametric two-tailed Wilcoxon matched-pairs signed-rank test for within-group comparisons (bars on top) and the nonparametric Kruskal–Wallis test for between-groups comparisons (bars on bottom). Differences were considered significant if *p* < 0.05.

**Fig. 2:**
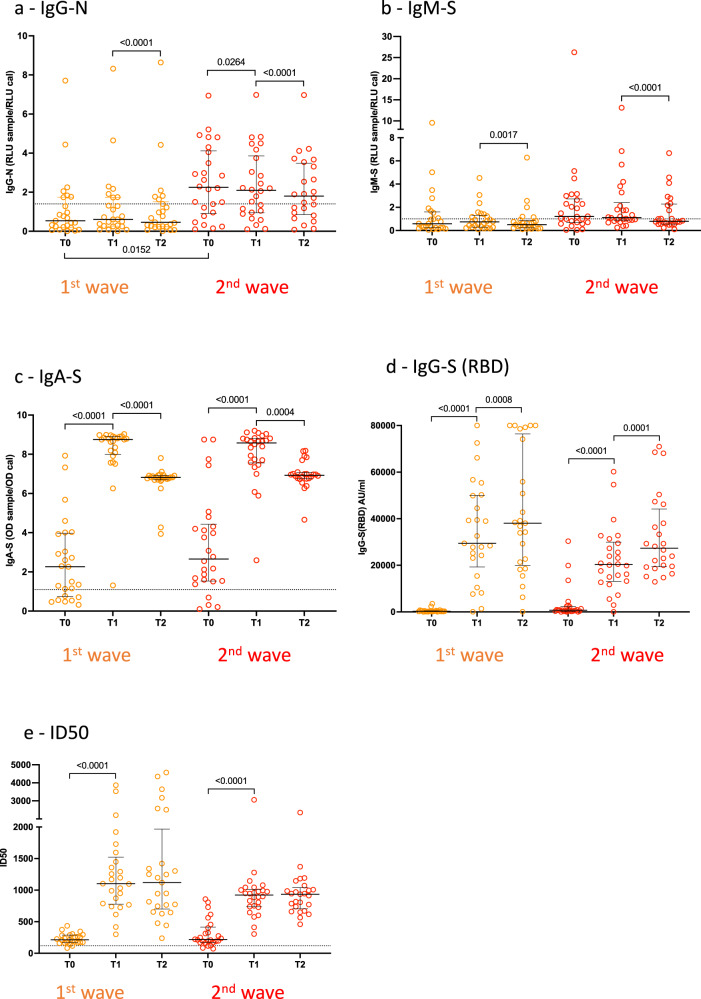
Analysis of the antibody response profile at the time of first vaccination (T0), second vaccination (T1), and 3 weeks after (T2) in subjects infected during the first (orange dots) and the second (red dots) COVID-19 wave. Median values with the interquartile range are displayed; the horizontal dot lines indicate cutoff value to discriminate positive and negative samples for each assay, according to the manufacturer’s instructions. **a** IgG for the SARS-CoV-2 nucleocapsid protein N (sample sizes: T0, T1, and T2 *n* = 25, for P.I. first wave, T0 and T1 *n* = 26, T2 *n* = 24 for P.I. second wave); **b** IgM for the spike glycoprotein (sample sizes: T0, T1, and T2 *n* = 25, for P.I. first wave, T0 and T1 *n* = 26, T2 *n* = 24 for P.I. second wave); **c** IgA for the spike glycoprotein (sample sizes: T0, T1, and T2 *n* = 25, for P.I. first wave, T0 and T1 *n* = 26, T2 *n* = 24 for P.I. second wave); **d** IgG for the receptor-binding domain (RBD) of the spike (sample sizes: T0, T1, and T2 *n* = 25, for P.I. first wave, T0 and T1 *n* = 26, T2 *n* = 24 for P.I. second wave); **e** neutralization assay, expressed as infectious dose (ID50) (sample sizes: T0, T1, and T2 *n* = 25, for P.I. first wave, T0 and T1 *n* = 26, T2 *n* = 25 for P.I. second wave). *P* values were calculated using the nonparametric two-tailed Wilcoxon matched-pairs signed-rank test for within-group comparisons (bars on top) and the nonparametric Kruskal–Wallis test for between-groups comparisons (bars on bottom). Differences were considered significant if *p* < 0.05.

**Fig. 3:**
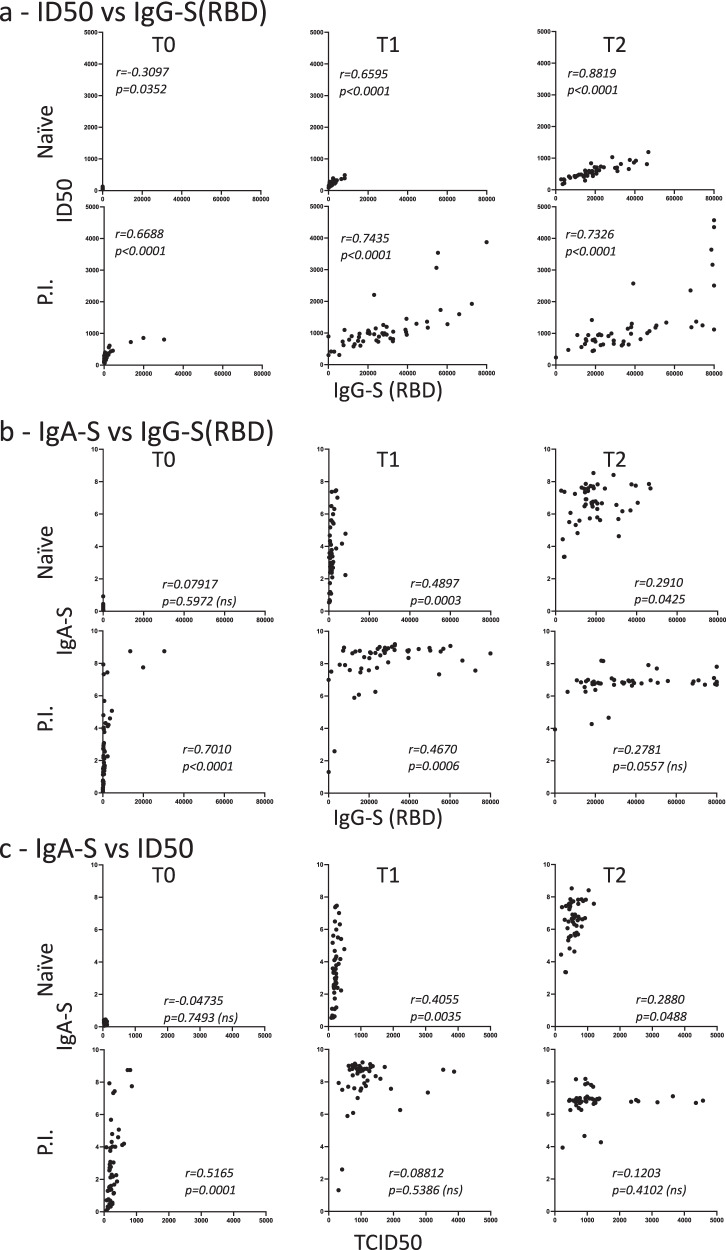
Analysis of the correlation at time of first vaccination (T0), second vaccination (T1), and 3 weeks after (T2) in naïve and previously infected (P.I.) recipients. **a** correlation between IgG-S(RBD) and neutralization (ID50) (sample sizes: T0 *n* = 48, T1 *n* = 50, T2 *n* = 49 for naïve, T0 and T1 *n* = 51, T2 *n* = 49 for P.I.); **b** correlation between IgG-S(RBD) and IgA (sample sizes: T0 *n* = 48, T1 *n* = 50, T2 *n* = 49 for naïve, T0 and T1 *n* = 51, T2 *n* = 49 for P.I.); **c** correlation between neutralization (ID50) and IgA-S (sample sizes: T0 *n* = 48, T1 *n* = 50, T2 *n* = 49 for naïve, T0 and T1 *n* = 51, T2 *n* = 49 for P.I.). The correlation was calculated using the two-sided Spearmen rank-correlation test.

**Fig. 4:**
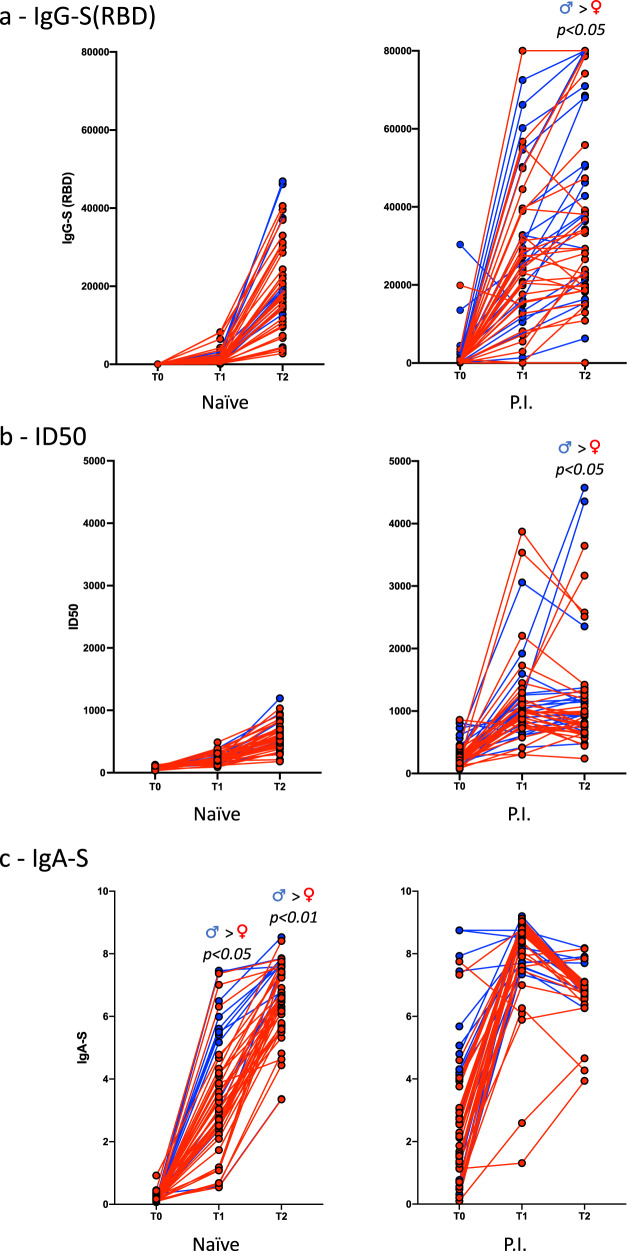
Antibody response in naïve and previously infected (P.I.) male (blue dots) and female (red dots) subjects following vaccination. **a** IgG-S(RBD) titers at the time of first vaccination (T0, *n*. males/females 12/36 for naïve, 19/32 for P.I.), second vaccination (T1, *n*. males/females 13/37 for naïve, 19/32 for P.I.), and 3 weeks after the boost (T2, *n*. males/females 13/36 for naïve, 19/30 for P.I.); **b** neutralization activity expressed as ID50 (*n*. males/females: T0 12/36, T1 13/37, T2 13/36 for naïve, T0 19/32, T1 19/32, T2 19/31 for P.I.); **c** IgA-S antibody titers (*n*. males/females: T0 12/37, T1 13/37, T2 13/36 for naïve, T0 19/32, T1 19/32, T2 18/31 for P.I.). *P* values were calculated using the Wilcoxon matched-pairs signed ranked test and considered significant if *p* < 0.05.

*P* values were calculated using the nonparametric two-tailed Wilcoxon matched-pairs signed-rank test for within-group comparisons and the nonparametric Kruskal–Wallis test for between-group comparisons, with *P* values adjusted for multiple comparisons using the Dunn’s test (Figs. 1 and  2), the two-sided Spearmen rank-correlation test (Fig. 3), the Wilcoxon matched-pairs signed ranked test (Fig. 4), and the chi-squared test (Table 1) using SPSS (version 22, SPSS Inc.) and Prism 9 (GraphPad Software, LLC). Significance was set at *p* < 0.05. Sample sizes for each test, each time point, and each subgroup of vaccine recipients are shown in Table 2.

**Table 1:** Characteristics of the study population.

	Naïve (50)	P.I. (51)	*p*
Males/Females	13/37	19/32	*p* = 0.23 (ns)
Age (yrs)	43 ± 13	46 ± 12	*p* = 0.21 (ns)
Fever after first dose (%)	7.7	0	*p* = 0.40 (ns)
Discomfort after the first dose (%)	26.9	22.2	*p* = 0.78 (ns)
Fever after the second dose (%)	38.5	33.3	*p* = 0.79 (ns)
Discomfort after the second dose (%)	53.8	66.7	*p* = 0.51 (ns)

P.I. previously infected, ns not significant.

**Table 2:** Antibody and neutralization assays (positives/total and percent).

Test	Time	Naïve	P.I.	First wave	Second wave
IgG-N	T0	0/48 (0%)	24/51 (47.1%)	7/25 (28%)	17/26 (65.4%)
	T1	0/50 (0%)	23/51 (45.1%)	7/25 (28%)	16/26 (61.5%)
	T2	0/49 (0%)	21/49 (42.9%)	6/25 (24%)	14/24 (58.3%)
IgM-S	T0	0/48 (0%)	24/51 (47.1%)	8/25 (32%)	16/26 (61.5%)
	T1	27/50 (54%)	23/51 (45.1%)	8/25 (32%)	15/26 (57.7%)
	T2	29/49 (59.2%)	15/49 (30.6%)	5/25 (20%)	10/24 (41.7%)
IgG-S(RBD)	T0	0/48 (0%)	46/51 (90.2%)	23/25 (92%)	23/26 (88.5%)
	T1	49/50 (98%)	50/51 (98%)	25/25 (100%)	25/26 (96.2%)
	T2	49/49 (100%)	49/49 (100%)	25/25 (100%)	24/24 (100%)
IgA-S	T0	0/49 (0%)	40/51 (78.4%)	18/25 (72%)	22/26 (84.6%)
	T1	44/50 (88%)	51/51 (100%)	25/25 (100%)	26/26 (100%)
	T2	49/49 (100%)	49/49 (100%)	25/25 (100%)	24/24 (100%)
ID50	T0	2/48 (4.2%)	46/51 (90.2%)	23/25 (92%)	23/26 (88.5%)
	T1	47/50 (94%)	51/51 (100%)	25/25 (100%)	26/26 (100%)
	T2	49/49 (100%)	50/50 (100%)	25/25 (100%)	25/25 (100%)

P.I. previously infected

## Results and discussion

Antibody levels were measured at three time points: prior to first vaccination (T0), prior to second vaccination (T1), and 3 weeks after the second vaccination (T2). As the BNT162b2 vaccine is expected to elicit only antibodies for the Spike glycoprotein, we also tested all subjects for the presence of IgG-nucleocapsid (IgG-N) antibodies to identify recipients with past undetected infection. The IgG-N antibody test is also a reliable marker of enduring immunity to SARS-CoV-2^21^ and as such can be used to monitor waning immunity. Since natural infection with SARS-CoV-2 is often followed by a rapid rise in IgG antibodies that can occur concomitantly or even before the appearance of IgM antibodies^14,22–24^ we tested whether a similar pattern would follow vaccination by measuring both IgG antibodies specific for the RBD of the spike protein (IgG-S(RBD)) and IgM spike-specific antibodies (IgM-S).

IgA have been implicated in protective immunity to SARS-CoV-2. During natural SARS-CoV-2 infection, IgA responses precede IgG responses^25–27^. Whether this is also the case after vaccination is unknown. We, therefore, tested all sera for the presence of IgG-N, IgM-S, IgG-S(RBD), and IgA-S as well as for the presence of virus-neutralizing activity as measured in a pseudovirus neutralization assay.

We enrolled 101 health-care workers with (P.I.) or without (naïve) preexisting immunity to SARS-CoV-2. Of the 51 P.I. vaccinees, 25 had been infected during the first wave and 26 during the second wave. All subjects received the first vaccine dose (BNT162b2 mRNA, Pfizer-BioNTech) in January 2021. The two groups were homogeneous in age and sex (Table 1).

IgG-N antibody testing was negative in all naïve recipients and positive in 24/51 (47%) P.I. recipients (Fig. 1a). A single subject, originally classified as naïve, who resulted negative at baseline but highly positive at T1 and T2 for the presence of IgG-N was excluded from the analysis. In the P.I group, at baseline, 7/25 (28%) and 17/26 (65%) of those who were infected during the first and second waves, respectively, were positive for IgG-N antibodies, consistent with a trend toward waning antibody titers in recipients infected during the first wave (Fig. 2a and Table 2).

IgM-S antibodies measured before vaccination (T0) showed a similar pattern with 8/25 (32%) and 16/26 (61%) testing positive at baseline in the first and second wave P.I. recipients, respectively (Fig. 2b and Table 2). Following vaccination, 27/50 (54%) naïve recipients became IgM-S positive after the first dose, and 29/49 (59%, 20 of whom were already positive after the first dose) were positive after the second dose with no significant increase in antibody titers compared to baseline (Fig. 1b and Table 2). In P.I. recipients from the first or second wave, there was no significant difference in the IgM-S response after vaccination (Fig. 2b and Table 2).

IgG-S(RBD) were detectable after the first vaccine dose in 49/50 (98%) naïve recipients but with very low titers that were boosted by the second vaccine dose resulting in a highly significant increase (*p* < 0.0001) (Fig. 1d and Table 2). Forty-six/51 (90%) P.I. recipients showed low IgG-S(RBD) titers at baseline (Fig. 1d and Table 2), and the first vaccine dose induced a strong increase in IgG-S(RBD) reaching median levels that were 22-fold higher than titers observed in naïve subjects after the first dose (naïve T1: median 1139, *n* = 50; P.I. T1: 25218, *n* = 51; *p* < 0.0001) and 1.9-fold higher when comparing P.I. and naïve recipients after the second dose (naïve T2: median 17913, *n* = 49; P.I. T2: median 33,296, *n* = 49), and it was 1.4 higher when comparing P.I. recipients after the first dose to naïve recipients after the second dose (naïve T2 median 17,913, *n* = 49; P.I. T1: median 25,218, *n* = 51). The second vaccine dose in P.I. recipients resulted in a significant 1.3-fold increase in antibody titers (P.I. T2: median 33,296, *n* = 49; P.I. T1: 25,218, *n* = 51; *p* < 0.0001). Comparison of P.I. recipients infected during the two waves showed no statistically significant differences in vaccine responses (Fig. 2d and Table 2), although an unexpectedly higher response to the first dose was observed in those infected during the first wave compared with those infected during the second wave, in agreement with a recent study^28^.

The pseudovirus neutralization assay (measured as ID50 values) showed in 47/50 (94%) naïve recipients a weak but positive score (>120 for ID50) after the first dose and in 49/49 (100%) after the second dose, with a highly significant (*p* < 0.0001) increase in neutralizing titers (Fig. 1e). In P.I. recipients, we observed an efficient boost of neutralizing antibodies after the first dose (*p* < 0.0001) and no further increase after the second dose (Fig. 1e), consistent with data reported by other authors^7–9,29^. Interestingly, we observed significantly higher neutralization titers at all time points in P.I. recipients compared to naïve (*p* < 0.0001 at T1, *p* = 0.0092 at T2). In addition, in P.I. recipients, neutralization titers were rapidly recalled by a single vaccine dose to levels higher than those observed in naïve recipients after the second vaccine dose (*p* = 0.0140, Fig. 1e), irrespective of waning antibody response (Fig. 2e).

A correlation analysis between the IgG-S(RBD) and the pseudovirus neutralization assay (ID50) confirmed a strong association between serum IgG-S(RBD) and neutralizing titers (Fig. 3a) consistent with other reports showing a major role played by RBD-specific antibodies in virus neutralization^30^. Results of the two waves P.I. cohorts also mirrored those obtained with the IgG-S(RBD) and evidenced a surprisingly, statistically significant (T1, *p* = 0.0290), higher virus neutralization response in P.I. recipients of the first wave compared with the second (Fig. 2e). These data are strongly suggestive of the persistence of memory B cell responses that can be rapidly recalled by a single vaccine dose after 9–10 months from primary infection even in the absence of detectable serum IgG-S(RBD) antibodies. It is also conceivable that natural infection with SARS-CoV-2 may prime the immune system to produce antibody specificities other than RBD that can be readily recalled by a single dose of vaccine.

The potential implication of cross-reactive immunity to other coronaviruses in the response to vaccination is supported by an unexpected feature that emerged from our data: the unconventional isotype pattern observed in both naïve and P.I recipients. In the naïve recipients, after the first dose, when the canonical primary immune response is expected to generate IgM first followed by IgG, only 27/50 (54%) recipients were positive for IgM-S with no further increase after the second dose (29/49; 59%), whereas 49/50 (98%) and 49/49 (100%) naïve recipients scored positive for IgG-S(RBD) after the first and second dose, respectively (Table 2). Twenty-three/50 (46%) naïve recipients showed an IgG-S(RBD) positive test but were negative for IgM-S, 27/50 (54%) were positive for both IgG-S(RBD) and IgM-S, and none were positive for IgM-S and negative for IgG-S(RBD) (Table 3). This isotype pattern is consistent with that of an anamnestic response sustained by memory B cells specific for spike epitopes shared with other common hCoVs^14,15^.

**Table 3:** Number and percent of IgM-S and IgG-S(RBD) positive subjects.

		Naïve			P.I.			First wave			Second wave		
IgM	IgG	T0	T1	T2	T0	T1	T2	T0	T1	T2	T0	T1	T2
**+**	**+**	0 (0%)	27 (54%)	29 (59.2%)	24 (47.1%)	22 (43.1%)	15 (30.6%)	8 (32%)	8 (32%)	5 (20%)	16 (61.5%)	14 (53.8%)	10 (41.7%)
**+**	**−**	0 (0%)	0 (0%)	0 (0%)	0 (0%)	1 (2%)	0 (0%)	0 (0%)	0 (0%)	0 (0%)	0 (0%)	1 (3.8%)	0 (0%)
**−**	**+**	1 (2.1%)	23 (46%)	20 (40.8%)	22 (43.1%)	28 (54.9%)	34 (69.4%)	15 (60%)	17 (68%)	20 (80%)	7 (26.9%)	11 (42.3%)	14 (58.3%)
**−**	**−**	47 (97.9%)	0 (0%)	0 (0%)	5 (9.8%)	0 (0%)	0 (0%)	2 (8%)	0 (0%)	0 (0%)	3 (11.5%)	0 (0%)	0 (0%)

P.I. previously infected

At baseline, IgA-S were detected in none of the naïve recipients, in 18/25 (72%) of the P.I. recipients who were infected during the first wave, and in 22/26 (84%) of those infected during the second wave (Fig. 1c and Table 2). The first vaccine dose resulted in a significant increase (*p* < 0.0001) in IgA-S titers in both naïve and P.I. recipients (Fig. 1c). The second dose further boosted the IgA-S titers in naïve recipients (*p* < 0.0001) but led to a significant reduction in P.I. recipients (*p* < 0.0001). The decline in IgA titers after the second vaccination in P.I. recipients was not related to the time from infection, as it was observed in both subjects infected during the first and second wave and most likely represents a response to the vaccination (Fig. 2c). A correlation analysis between the IgA-S and IgG-S(RBD) titers at baseline and T1 and T2 revealed in naïve subjects the appearance of high IgA titers after the first vaccine dose followed by a significant increase in IgG-S(RBD) titers only after the second dose (Fig. 3b). In P.I. recipients, the first and second dose boosted both IgA-S and IgG-S(RBD) titers (Fig. 3b). We did not observe a correlation between IgA-S titers and virus neutralization titers (ID50) both in naïve (Fig. 3c, T1) and P.I. recipients (Fig. 3c, T0). On the other hand, the early increase in IgA after the first dose that preceded the increase in IgG-S(RBD) titers after the second vaccine dose is consistent with what has been observed during natural SARS-CoV-2 infection where IgAs precede IgGs^25–27^. The presence of IgA-S in a significant proportion of P.I. recipients at baseline is also consistent with the slower waning of IgA compared to IgG observed in convalescent patients^31^. An alternative explanation, although unlikely, is the possibility of increased assay sensitivity for IgA, which cannot be formally ruled out.

We next examined the influence of sex in the IgG-S(RBD) and IgA-S responses to vaccination. In the naïve recipients, there were no differences in the kinetic and size of IgG-S(RBD) responses between males and females. In contrast, in the P.I. group, males responded to the vaccine by producing higher titers of IgG-S(RBD) than females (*p* < 0.05) (Fig. 4a). This result is different from some very recent findings reporting a higher IgG response in women compared to men^28,32^, which could be due to the characteristics of the population analysed, our being composed of healthy and relatively young health-care workers (Table 1).

This difference was mirrored by the neutralization assay, which showed higher neutralizing titers in males than in females (Fig. 4b). The differences between the two groups were significant in male and female naïve IgA-S responses at T1 (*p* < 0.05) and T2 (*p* < 0.01) (Fig. 4c).

We observed no significant correlation between antibody titers/neutralizing activity and symptom severity in P.I. subjects, possibly due to the young population analysed (median 45 years, subjects mostly reported weak symptomatology or were asymptomatic). Similarly, we did not find any significant correlation with post-vaccination systemic reactions (i.e., fever, malaise) in both naïve and P.I. subjects after the first or second dose of vaccine.

Our findings expand on previous studies that indicated higher levels of anti-S antibodies at baseline and after a single mRNA vaccine dose in P.I. individuals compared with those without prior infection, suggesting that a second vaccine dose does not offer P.I. recipients a substantially greater benefit over a single dose in terms of antibody neutralization. The availability of two subgroups of recipients who had been infected during the first and second waves of the pandemic in Italy gave us the additional opportunity to evaluate the effects of one dose versus two doses of vaccination in the context of a past-waning immunity and compare it with that of recent-active immunity. The significantly lower IgG-N and IgG-S(RBD) antibody titers observed at baseline in the first wave P.I. vaccinees compared to the second wave P.I. vaccinees (Fig. 2a, d) gave us confidence that the two cohorts were in two different stages of postinfection immunity

When IgA-S antibody titers were considered, no substantial differences were observed between the two cohorts at baseline, suggesting a slower rate of IgA decline, at least in our cohort, than that reported in the literature. Although we cannot formally exclude the possibility that the observed rates of IgA positivity may be due to the specificity and sensitivity of the assay, the possibility that in health-care workers repeated exposures to the virus may maintain a low but consistent level of mucosal, as well as systemic, IgA response should be considered.

We observed a surprising rapid recall of high IgG-S(RBD) and virus-neutralizing titers observed in first wave P.I. recipients even in individuals with absent or very low serum IgG-S(RBD) levels. The response observed in the first wave P.I. recipients was significantly higher than that observed in the second wave P.I. recipients, probably as a result of the persistence of memory B cell responses that can be rapidly recalled by a single dose and consistent with recent data on the appearance in convalescent patients of memory B cells with a turnover time of 6 months that express antibodies with increased somatic hypermutations, neutralizing breadth, and potency^33^. We speculate that the Spike-specific antibody repertoire generated by natural infection and boosted by the first vaccination may be broader than that induced by vaccination in naïve individuals, thus generating more antibody specificities with neutralization potential.

Our study did not address the epitope specificities of vaccination-induced antibodies, and additional studies addressing the fine specificities of vaccine-induced antibodies are warranted.

A priming effect of previous exposures to common hCoVs on the immune response to the vaccine is suggested by our findings in the cohort of naïve recipients showing IgG-S(RBD) antibodies in the absence of IgM-S antibodies. Antibodies that cross-neutralize SARS-CoV-1, SARS-CoV-2, and other coronaviruses have been described that bind conserved epitopes of the hACE2 binding site showing extensive conservation among the SARS-like coronaviruses^34^. In COVID-19 patients the ability of rapidly switching the antibody response from IgM/IgA to IgG is clearly associated with better disease outcome^35^. Here we speculate that the appearance of IgG in the absence of IgM in naïve recipients after vaccination is a hallmark of vaccine efficacy. It remains to be determined whether this phenomenon is due to the capacity of the BTN162b2 mRNA vaccine to elicit cross-reactive antibodies or to some other yet unknown factor.

Our findings on the more rapid and potent IgA response compared with IgG responses to the first vaccine dose parallel those in SARS-CoV-2 infected patients, where IgA antibodies that bind to SARS-CoV-2 are produced rapidly after infection and remain elevated in the plasma for at least 40 days after the onset of symptoms^36^. We did not observe a significant association between serum IgA and virus-neutralizing activity post-vaccination, which, in contrast, has been reported in COVID-19 patients^30^. However, it is plausible that the types of IgA antibodies elicited by intramuscular vaccination may differ from the compartmentalized, mucosal immune response to natural infection. Accordingly, SARS-CoV-2 specific plasma IgA monomers have been shown to be two times less potent than IgG equivalents and, in contrast, IgA dimers, the principal type of antibody in the nasopharynx, are 15 times more potent against the same target as IgA monomers^36^. On the other hand, emerging vaccine correlates of immunity point to important roles of both virus-neutralizing and Fc receptors functions of antibodies in protection from infection and disease where neutralization represents the first line of defense and Fc receptor function may provide a second line of defense deeper in the respiratory tract^35^. Whether vaccine-induced serum monomeric IgA play a role in SARS-CoV-2 immunity by triggering the IgA Fc receptor (FcαRI/CD89) on phagocytes remains speculative and deserves further investigation. Our data clearly show a rapid and potent induction of IgG-S(RBD) after a dose of vaccine in individuals with past-waning antibody titers. Although a clear correlate of protection from COVID-19 has not yet been identified, the findings by Zohar and colleagues that, notwithstanding equivalent IgM and IgA immunity to the virus observed in different disease severity levels, rapid and potent IgG class switching is associated with survival^35^ provide an additional argument in support of the use of a single-dose vaccine regimen in individuals with prior SARS-CoV-2 infections.

Limitations of our study include the sample size, the use of only one type of vaccine (Pfizer-BioNTech BNT162b2), the lack of information on T-cell responses, and neutralization response against emerging SARS-CoV-2 variants of concern.

In conclusion, our data show that: (1) immunologically naïve recipients react to the first dose of vaccine with a low-titer IgG-S(RBD) response that is then boosted by the second dose; (2) in P.I. recipients one dose of vaccine is sufficient to induce antibody titers that are higher than those observed in naïve recipients after the second vaccine dose; (3) there is a good correlation between IgG-S(RBD) titers and virus-neutralizing titers, confirming that the IgG-S(RBD) testing is a proxy for virus neutralization; (4) recipients who were infected with SARS-CoV-2 during the first pandemic wave exhibit a rapid response to vaccination measured as both IgG-S(RBD) binding titers and virus-neutralizing titers; (5) in 46% of naïve recipients, IgG-S(RBD) appear in the absence of IgM-S as if the response to vaccination was influenced by previous antigen exposures; (6) in naïve recipients IgA-S appear before IgG-S(RBD) after the first vaccine dose and are further boosted by the second dose; (7) in P.I. recipients, IgA show a rapid, high titer response to the fist vaccine dose that is followed by a decline with the second dose; (8) there is no correlation between IgA-S antibody titers and virus neutralization; (9) neutralization titers and IgG-S(RBD) were higher in P.I. male recipients compared to female but not in naïve recipients.

### Reporting Summary

Further information on research design is available in the Nature Research Reporting Summary linked to this article.

## Supplementary information

Reporting Summary

## Data Availability

Source data is available via Figshare at 10.6084/m9.figshare.14776212
