# Supplementary material for: Serology study after BTN162b2 vaccination in participants previously infected with SARS-CoV-2 in two different waves versus naïve
Source: Commun Med (Lond). 2021 Oct 13;1:38. doi: 10.1038/s43856-021-00039-7 (PMC9053253; doi:10.1038/s43856-021-00039-7)
Supplement: Supplementary file 1 — Reporting Summary [file 43856_2021_39_MOESM1_ESM.pdf]

nature research

Corresponding author(s): Donato Zipeto

Last updated by author(s): Sep 14, 2021

Reporting Summary

Nature Research wishes to improve the reproducibility of the work that we publish. This form provides structure for consistency and transparency in reporting. For further information on Nature Research policies, see our Editorial Policies and the Editorial Policy Checklist.

Statistics

For all statistical analyses, confirm that the following items are present in the figure legend, table legend, main text, or Methods section.

n/a	Confirmed	
☐	☒	The exact sample size (*n*) for each experimental group/condition, given as a discrete number and unit of measurement
☐	☒	A statement on whether measurements were taken from distinct samples or whether the same sample was measured repeatedly
☐	☒	The statistical test(s) used AND whether they are one- or two-sided<br>*Only common tests should be described solely by name; describe more complex techniques in the Methods section.*
☐	☒	A description of all covariates tested
☒	☐	A description of any assumptions or corrections, such as tests of normality and adjustment for multiple comparisons
☒	☐	A full description of the statistical parameters including central tendency (e.g. means) or other basic estimates (e.g. regression coefficient) AND variation (e.g. standard deviation) or associated estimates of uncertainty (e.g. confidence intervals)
☐	☒	For null hypothesis testing, the test statistic (e.g. *F*, *t*, *r*) with confidence intervals, effect sizes, degrees of freedom and *P* value noted<br>*Give P values as exact values whenever suitable.*
☒	☐	For Bayesian analysis, information on the choice of priors and Markov chain Monte Carlo settings
☒	☐	For hierarchical and complex designs, identification of the appropriate level for tests and full reporting of outcomes
☒	☐	Estimates of effect sizes (e.g. Cohen's *d*, Pearson's *r*), indicating how they were calculated

Our web collection on statistics for biologists contains articles on many of the points above.

Software and code

Policy information about availability of computer code

Data collection	No custom algorithms or software used.
Data analysis	All statistical analyses were conducted using SPSS (version 22, SPSS Inc.) and Prism 9 (GraphPad Software, LLC)

For manuscripts utilizing custom algorithms or software that are central to the research but not yet described in published literature, software must be made available to editors and reviewers. We strongly encourage code deposition in a community repository (e.g. GitHub). See the Nature Research guidelines for submitting code & software for further information.

Data

Policy information about availability of data

All manuscripts must include a data availability statement. This statement should provide the following information, where applicable:

- Accession codes, unique identifiers, or web links for publicly available datasets
- A list of figures that have associated raw data
- A description of any restrictions on data availability

The authors declare that the data supporting the findings of this study are available within the paper.

Field-specific reporting

Please select the one below that is the best fit for your research. If you are not sure, read the appropriate sections before making your selection.

☒ Life sciences ☐ Behavioural & social sciences ☐ Ecological, evolutionary & environmental sciences

For a reference copy of the document with all sections, see nature.com/documents/nr-reporting-summary-flat.pdf

Life sciences study design

All studies must disclose on these points even when the disclosure is negative.

Sample size	We did not perform statistical analyses to predetermine sample sizes. We collected 50 naïve subjects; 25 infected during the first wave in March-May 2020, 26 during the second wave in October 2020-January 2021 who received their first vaccine dose (BNT162b2 mRNA, Pfizer-BioNTech) in January 2021.
Data exclusions	A single subject, originally classified as naïve, who resulted negative at baseline but highly positive at T1 and T2 for the presence of IgG-N was excluded from the study
Replication	Precision and reproducibility experiments were conducted in duplicate.
Randomization	Our study is an observation study, so no randomization is needed here.
Blinding	Serum extraction and antibody detection were performed independently by researchers blind to samples information, data analysis were performed by two trained researchers.

Reporting for specific materials, systems and methods

We require information from authors about some types of materials, experimental systems and methods used in many studies. Here, indicate whether each material, system or method listed is relevant to your study. If you are not sure if a list item applies to your research, read the appropriate section before selecting a response.

Materials & experimental systems

n/a	Involved in the study	
☐	☒	Antibodies
☒	☐	Eukaryotic cell lines
☒	☐	Palaeontology and archaeology
☒	☐	Animals and other organisms
☐	☒	Human research participants
☒	☐	Clinical data
☒	☐	Dual use research of concern

Methods

n/a	Involved in the study	
☒	☐	ChIP-seq
☒	☐	Flow cytometry
☒	☐	MRI-based neuroimaging

Antibodies

Antibodies used	*Describe all antibodies used in the study; as applicable, provide supplier name, catalog number, clone name, and lot number.*
Validation	The antibody was only used for the application as indicated and organisms verified by the manufactures.

Human research participants

Policy information about studies involving human research participants

Population characteristics	Male and Female healthcare workers, 50 Naïve (37F, 13M) aged 23-68 years old and 51 (32F, 19M) aged 23-59 years previously infected were recruited in the Azienda Ospedaliera Universitaria Integrata of Verona and IRCCS Sacro Cuore Don Calabria Hospital, Negrar (Verona).
Recruitment	We enrolled 101 healthcare workers with and without pre-existing immunity for SARS-CoV-2 (naïve: 50 subjects; 25 infected during the first wave in March-May 2020, 26 during the second wave in October 2020-January 2021) who received their first vaccine dose (BNT162b2 mRNA, Pfizer-BioNTech) in January 2021.
Ethics oversight	Ethics Committee for Clinical Trials (CESC) of the Provinces of Verona and Rovigo based at AOUI of Verona

Note that full information on the approval of the study protocol must also be provided in the manuscript.
